# Rabies of canid biotype in wild dog (*Lycaon pictus*) and spotted hyaena (*Crocuta crocuta*) in Madikwe Game Reserve, South Africa in 2014–2015: Diagnosis, possible origins and implications for control

**DOI:** 10.4102/jsava.v89i0.1517

**Published:** 2018-04-26

**Authors:** Claude T. Sabeta, Drienie D. Janse van Rensburg, Baby Phahladira, Debra Mohale, Robert F. Harrison-White, Carlien Esterhuyzen, June H. Williams

**Affiliations:** 1 Onderstepoort Veterinary Institute, OIE Rabies Reference Laboratory, South Africa; 2 Department of Paraclinical Sciences, University of Pretoria, South Africa; 3 Predator Research Project, Madikwe, South Africa; 4 Madikwe Conservancy Private Game Reserve, North West province, South Africa

## Abstract

Both domestic and wild carnivore species are commonly diagnosed with rabies virus (RABV) infection in South Africa. Although the majority of confirmed rabies cases in wild carnivore species are reported from the yellow mongoose (*Cynictis penicillata*), the rest are from other wild carnivores including the highly endangered wild dog (*Lycaon pictus*). Lyssavirus infection was confirmed in two wild dogs and a spotted hyaena (*Crocuta crocuta*) in the Madikwe Game Reserve, North West province in South Africa, in 2014 and 2015, using a direct fluorescent antibody test and immunohistochemistry. There had been no new wild dog introductions to the Madikwe Game Reserve for many years and the wild dogs were last vaccinated against rabies approximately 11 years prior to the incident. The first euthanised wild dog was the last surviving of a break-away pack of 6, and the second was the last of a larger pack of 18, the rest of which died with no carcasses being found or carcasses too decomposed for sampling. Subsequent antigenic typing of the lyssaviruses indicated that they were canid RABVs. The RABVs originating from 22 wild carnivore species, 7 dogs, and a caprine, mostly from the North West province, were genetically characterised by targeting a partial region of the nucleoprotein gene. The nucleotide sequence analyses of these viruses and two previously characterised RABVs confirmed that the outbreak viruses were also canid rabies, phylogenetically clustering with virus isolates originating from black-backed jackals recovered between 2012 and 2015 from the North West province, and domestic dogs from neighbouring communal areas. The source(s) of the mortalities and possible reservoir host(s) for the virus could only be speculated upon from data on specific predator numbers, movements and behaviour, kills, park management and the changing environmental ecology, which were monitored closely in Madikwe over several years. The most likely rabies sources were from boundary fence contacts between wild carnivores within the park, with domestic dogs or cats and/or naturally occurring wild carnivores outside the park. The associated risk of zoonotic infection and threat to important and endangered predators may be mitigated through regional rabies control primarily in domestic dogs and cats, as well as by preventative vaccination of at-risk park employees and their pets. The importance of ongoing prophylactic rabies protection by regular vaccination of highly endangered wildlife carnivores and the submission of carcasses for rabies diagnosis of any wild or domestic animals behaving uncharacteristically or found dead is emphasised.

## Introduction

The wild dog (*Lycaon pictus*), also referred to as the African painted dog, is considered one of the most endangered wildlife carnivore species in sub-Saharan Africa (Woodroffe & Sillero-Zubiri 2012), and although once widespread, is now extinct in 19 of the 34 countries in which it once existed. The majority of the wild dog population now occurs in the arid zones and in the savannah in southern and south-eastern regions of the African continent. Under ideal conditions, African wild dog populations are able to grow relatively quickly. The capacity of wild dogs for long-range dispersal implies that their populations sometimes reappear unexpectedly and grow rapidly, as shown by natural recoveries in Samburu and Laikipa districts in Kenya (Woodroffe 2011), in the Save Conservancy in Zimbabwe (Pole 2000) and the Serengeti ecosystem (Marsden, Wayne & Mable 2011). Wild dog populations have since 1990 been classified by the International Union for Conservation of Nature (IUCN) as endangered and declining in numbers. A mere 6600 individuals in 39 subpopulations, of which only 1400 were mature individuals, were estimated in 2012 to remain in the wild, with causes of decline given as habitat fragmentation, conflict with human activities and infectious diseases (IUCN 2012). Whereas this decline is generally attributed to habitat loss, other detrimental factors and threats reported include human persecution, accidental capture in wire snares, loss of prey, predation by lions and perhaps competition with larger carnivores such as spotted hyaenas (Darnell et al. 2014). All the causes associated with human encroachment on the African wild dog have not ceased and are unlikely to be reversed across the majority of this species’ historical range.

Increasingly, spillover of canine pathogens such as rabies virus (RABV) (Kat et al. 1995) and canine distemper virus (CDV) (Alexander et al. 1996) into susceptible wildlife significantly reduces these wild carnivore populations (Berentsen et al. 2013; Cleaveland et al. 2007; Gowtage-Sequeira et al. 2009). RABV also poses a threat to humans (Hampson et al. 2008, 2009; Knobel et al. 2005) and is therefore a disease of paramount importance in Africa, given the extensive circulation and repeated spillovers of infection from the main vector and reservoir species, the domestic dog (Bingham 2005; Lembo et al. 2008). This occurs especially in circumstances where the domestic dog population density is more than 5 and preferably more than 11 per square km, and where they are free-roaming and not vaccinated (Lembo et al. 2008). In many such circumstances, dog rabies outbreaks led to new epidemiological cycles particularly in wildlife species (Lembo et al. 2008). This view of the role of population density has been challenged (Morters et al. 2013), however, and concluded to be more complex, likely differing between species, and complicated by human–dog interactions and movements, especially those in the face of interventions, which may be instigated during rabies outbreaks, such as culling, vaccination and sterilisation (Lembo et al. 2010).

Rabies contributed significantly to wild dog mortalities in the 1990s in the Serengeti, in Tanzania (Cleaveland & Dye 1995; Gascoyne et al. 1993), in the Masai Mara in Kenya (Kat et al. 1996), as well as in the Ethiopian wolf (*Canis simensis*) in the Ethiopian Bale mountains (Sillero-Zubiri, King & Mcdonald 1996). A study over 18 years (1989–2006) in Botswana (Moagabo et al. 2009) of 4306 positive rabies samples showed that over 60% of the 343 wildlife cases were jackal, with 2 from wild dogs. Mitigating such disease threats is challenging, partly because of uncertainty regarding disease dynamics making it difficult to identify the best management approaches. These include identifying reservoir or maintenance hosts (Hampson et al. 2009; Haydon et al. 2002), their population density (Morters et al. 2013) and the susceptibility of various wildlife carnivore species to rabies. Spotted hyaenas, for example, were found to be repeatedly infected with increasing age but not susceptible to the specific rabies strain circulating amongst them in the Serengeti (East et al. 2001). In contrast, a virulent strain reportedly caused mortality especially in aged spotted hyaenas in the Kalahari (Mills 1990). Also, infrequent contact between individual spotted hyaenas in a clan system of low density might tend to delay rabies spread, whereas the frequent especially mouth-to-mouth contacts between wild dog individuals within a pack because of their highly social nature would allow rapid intra-pack spread of rabies (Mills 1993). During a six-week period in 1989 in the Masai Mara of Kenya, 21 members of a wild dog pack numbering 23 died: brains examined histologically from two of these had Negri bodies and another brain tested by fluorescent antibody test (FAT) was positive for rabies (Kat et al. 1995).

According to Haydon et al. (2002) a reservoir comprises one or more epidemiologically connected populations or environments in which a pathogen can be permanently maintained and from which infection is transmitted to the defined target population. A source population may be a maintenance or a non-maintenance host, which transmits the pathogen to the target population. A number of non-maintenance populations could constitute a maintenance community. This has replaced a previous definition of a reservoir host as being one in which the relevant pathogen is non-pathogenic to the reservoir host (Haydon et al. 2002). Bingham (2005) differentiated between maintenance, which included the idea of indefinite transmission of infection in a local population, and persistence, which implied long-term continuity.

Serological evidence lends support to exposure of some wildlife species to a plethora of pathogens. For example, it was shown that spotted hyaenas in East Africa, black-backed jackals and captive cheetahs in Namibia, as well as the African wild dog, were exposed to bacterial pathogens such as *Ehrlichia canis* (Stevenson-Hamilton 1939) and to viral pathogens such as RABV and CDV (Bellan et al. 2012; Creel et al. 1997; East et al. 2001; Kat et al. 1996; Prager et al. 2012; Thalwitzer et al. 2010; Woodroffe et al. 2012). The consequences of such multi-host pathogen exposures, however, are largely unknown (Bellan et al. 2012; Creel et al. 1997; East et al. 2001; Gowtage-Sequeira et al. 2009; Kat et al. 1996; Prager et al. 2012; Thalwitzer et al. 2010; Woodroffe et al. 2012). Wild dogs and black-backed jackals were experimentally infected with *E. canis* from domestic dogs. Clinical signs were noted only in the wild dogs despite both species showing morulae in peripheral blood smears, and the infection was transmissible back to domestic dogs from both species (Van Heerden 1979). *Babesia canis* was similarly experimentally transmitted from domestic dog to these two species, with neither species showing clinical signs despite both showing parasitic trophozoites in their blood smears, and transmission back to domestic dogs was successful from both species (Van Heerden 1980). Black-backed jackals in Etosha National Park, Namibia, commonly have serum antibodies that are indicative of past exposure to CDV, with increasing numbers of animals positive with age without showing clinical evidence of disease (Bellan et al. 2012). A few individuals (7 of 81 tested, 9%) also had RABV antibodies and three of these did not develop clinical signs when monitored for more than a year (Bellan et al. 2012). Because rabies is generally thought to be a fatal disease when transmitted via bites, development of antibodies in non-affected animals is unusual, and in one study, it only occurred in one of two clinically rabid jackal (Bellan et al. 2012). Berentsen et al. (2013) found that 8 of 20 lions tested serologically in Zambia were positive for RABV neutralising antibodies without showing clinical signs. Patterns of seroprevalence in different age groups can help establish whether animals are facing a chronic disease threat or whether seropositive animals simply represent evidence of past epidemics or transfer of maternal antibodies (Bellan et al. 2012; Ginsberg & Woodroffe 1986). Primary subclinical exposure could also occur after ingestion of the virus and not via bite transmission.

Serological evidence of exposure to RABV and CDV in domestic animals and wildlife demonstrated that domestic dogs were maintenance hosts for RABV and that exposure was more likely in this species than in the wild carnivores (Prager et al. 2012). Mathematical modelling provided arguments that the side-striped jackal, *Canis adustus*, occurs at densities too low to maintain (i.e. facilitate indefinite local transmission) RABV without repeated introduction of the virus from domestic dogs (Rhodes et al. 1998). However, Bingham (2005), referring to domestic dog and jackal populations in Zimbabwe, suggested that RABV can persist in jackal populations independent of spillover from domestic dogs. In the western region of Limpopo province in northern South Africa, a genetic RABV cluster specific to black-backed jackals was found, which persisted for 5 years and was distinct from those found in domestic dogs in other regions surveyed in Limpopo. This suggested that jackal may sustain their own RABV cycle (Zulu, Sabeta & Nel 2009).

The potential threat of zoonotic disease transfer is increasing with the expanding global human population, along with increasing global tourism, the decrease in natural habitat areas for wildlife and the increasing human–wildlife interface in and around game parks. There is therefore not only the need to adopt appropriate countermeasures for the protection of endangered wildlife species such as wild dogs but also the associated need to diminish opportunities for zoonotic disease transfer to humans.

Conservation of wild carnivores has been successful by means of the application of mass domestic dog parenteral vaccination. Data from the field demonstrated that annual vaccination of at least 67% of the domestic dog population resulted in the control of rabies in Ngorongoro in Tanzania (Fitzpatrick et al. 2012). Oral rabies vaccination (ORV) was initially successful in eliminating the disease in certain wildlife reservoirs such as foxes in Europe (Aubert et al. 1994). Elsewhere in Africa, effective vaccination with an attenuated oral RABV vaccine, Street Alabama Gif (SAG-2; Virbac, France), has replaced parenteral vaccination and has shown great promise in the African wild dog (Knobel, Du Toit & Bingham 2002) and in two species of jackals (*C. adustus* and *Canis mesomelas*) (Bingham et al. 1999a). Oral vaccination of wild dogs using chicken heads baited with SAG-2 elicited potentially protective neutralising antibody levels that were detected 31 days post-vaccination (Knobel et al. 2002) and this approach may be key to protecting free-ranging wild dogs against RABV infection. More recently, field trials using goat meat demonstrated that Ethiopian wolves can be vaccinated using oral rabies vaccines (SAG-2) (Sillero-Zubiri et al. 2016).

A study was conducted by Van Heerden et al. (2002) using commercial vaccines intended for use in domestic dogs for rabies, canine distemper and canine parvovirus, in 11-month-old captive wild dogs, with the view to monitoring the safety and efficacy in inducing neutralising antibodies. Administration in four dogs each of either inactivated parenteral or oral live rabies vaccines (given three times) initially resulted in seroconversion in seven of the eight dogs (Van Heerden et al. 2002). These titres, however, dropped drastically within 100 days. It was concluded that the vaccines were safe to use in healthy subadult wild dogs and that a vaccination protocol in free-ranging wild dogs should at least incorporate booster vaccinations against rabies 3–6 months after the first inoculation.

An outbreak of rabies occurred in 2000 in a pack of wild dogs in the Madikwe Game Reserve, South Africa (Hofmeyr et al. 2004). This was the second outbreak of rabies in wild dogs following their re-introduction to the Reserve in 1995, the first having been recorded in 1997. The outbreak in question resulted in the death or disappearance of ten out of twelve 8-month-old pups. Because jackals (*C. mesomelas*) were regarded as the principal host species of rabies in the area, it was assumed that the jackal cycle was the source of the infection. Whereas the pups had not been vaccinated, the five adults, all of which survived, had each been vaccinated by the parenteral route at least twice and all had significant rabies serum–neutralising antibodies. This observation showed that multiple vaccinations against rabies were effective in protecting wild dogs against natural challenge, lending support to findings from other studies in captive wild dogs (Knobel, Liebenberg & Du Toit 2003).

Recently, wild carnivore species are increasingly being devastated by emerging and re-emerging zoonotic diseases. This report describes recent laboratory-confirmed RABV infections in two wild dogs and a hyaena in the Madikwe Game Reserve (North West province of South Africa). Through antigenic and genetic characterisation as well as background Madikwe predator and environmental data, the report attempts to identify the possible sources of infection in the park.

Madikwe Game Reserve is situated in the North West province of South Africa and is bordered on the west by Botswana and was announced in 1991. It is spread over 680 km^2^, which has been expanded to 750 km^2^ by newly incorporated private reserves. It was previously degraded farm land and has recovered as a result of sustainable use by a diversity of wildlife including 66 large animal species. The perimeter is 150 km of electrified fencing. The geography is large open grassland plains, woodlands with rocky outcrops and single mountains (http://www.madikwegamereserve.co.za).

## Description of cases

**FIGURE 1:**
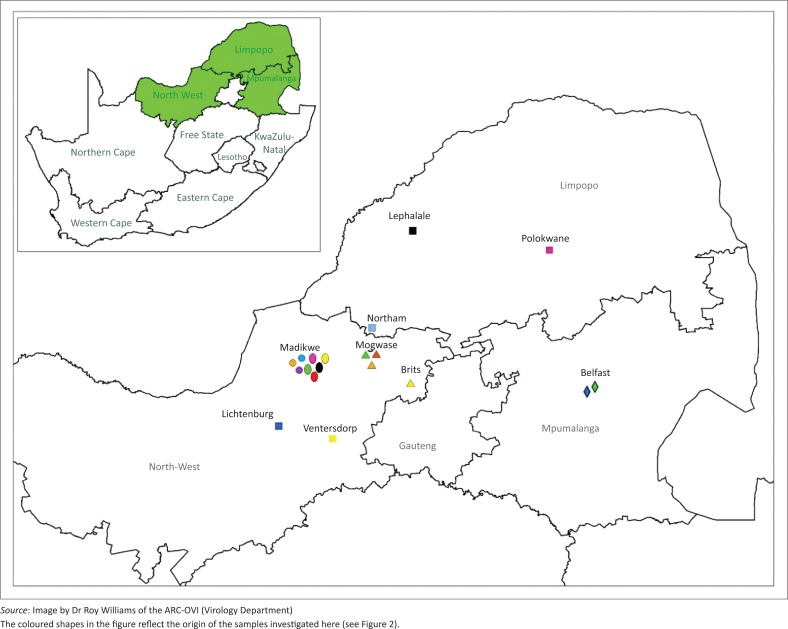
Map showing the North West province of South Africa.

On 26 December 2014, a wild dog was found biting at light bulbs in a tourist lodge in Madikwe Game Reserve (Figure 1). It was subsequently shot dead by a park official. This animal was the last of a breakaway pack of six dogs, the rest of which disappeared. The five people who handled the carcass were subsequently each administered VeroRab^®^ (Sanofi Pasteur, Marcy-l’Étoile, France) vaccination as prescribed by the manufacturer. A month later, a second wild dog, the last surviving member of a larger pack of 18 wild dogs, which had also presumably succumbed to rabies, had been showing severely deranged behaviour for 3–4 days at the same tourist lodge in Madikwe. It had chased staff and guests around the lodge, torn sofas to shreds, chewed on furniture and roamed inside the lodge buildings, fortunately without direct human contact, until it was shot at the nearby waterhole on 26 January 2015. Subsequent to this, a carcass of a spotted hyaena found dead at a waterhole near the tourist lodge on 27 February 2015 was submitted for testing.

### Virus investigation

Half of the brain from the carcasses of the first male wild dog (Agricultural Research Council – Onderstepoort Veterinary Institute [ARC-OVI] reference number# 889/14, i.e. sample number 889 of 2014), the second wild dog (ARC-OVI reference number# 66/15) and the spotted hyaena (ARC-OVI reference number# 113/15) were submitted on ice to the OIE Rabies Reference Laboratory at the ARC-OVI to exclude lyssavirus infection. Immunohistochemistry (IHC) was carried out at the Section of Pathology, Department of Paraclinical Sciences at the Faculty of Veterinary Science, University of Pretoria, Onderstepoort on formalinised, wax-embedded brain tissues from the first wild dog and the hyaena. On receipt of the specimens at ARC-OVI, composite brain smears were prepared and subjected to the direct fluorescent antibody test (dFAT) as described previously (Dean, Abelseth & Ataanasiu 1996), and counterstained with Evans Blue (at 0.00125% concentration). In brief, the smears were acetone-fixed and stained with a polyclonal anti-lyssavirus fluorescein isothiocyanate–labelled conjugate (ARC-OVI, Pretoria) and then viewed under ultraviolet fluorescence. The carcass of the second wild dog showed advanced autolysis; however, brain specimens for dFAT were taken and performed as for the first. Rabies immunohistochemical labelling using the immunoperoxidase (IMP) test was performed in parallel on brain tissues of two of the Madikwe animals (the first wild dog and hyaena). Sections of cerebellum, cerebrum, hippocampus, midbrain and salivary glands were labelled and evaluated using a polyclonal antibody (prepared by the late Dr Ken Charlton: Agriculture Canada: Animal Diseases Research Institute: Nepean Polyclonal Rabbit Anti-Rabies Antibody) at 1:500 dilution using a standard published method (Haines & Chelack 1991) and was adapted by Pashwane and Clift of the Veterinary Faculty’s Pathology IHC Laboratory (2010). Subsequent to the positive dFAT and IMP tests, the lyssaviruses were then typed with a panel of 16 anti-nucleoprotein monoclonal antibodies (provided by the Canadian Food Inspection Agency, Canada), as described previously (Ngoepe et al. 2014).

**FIGURE 2:**
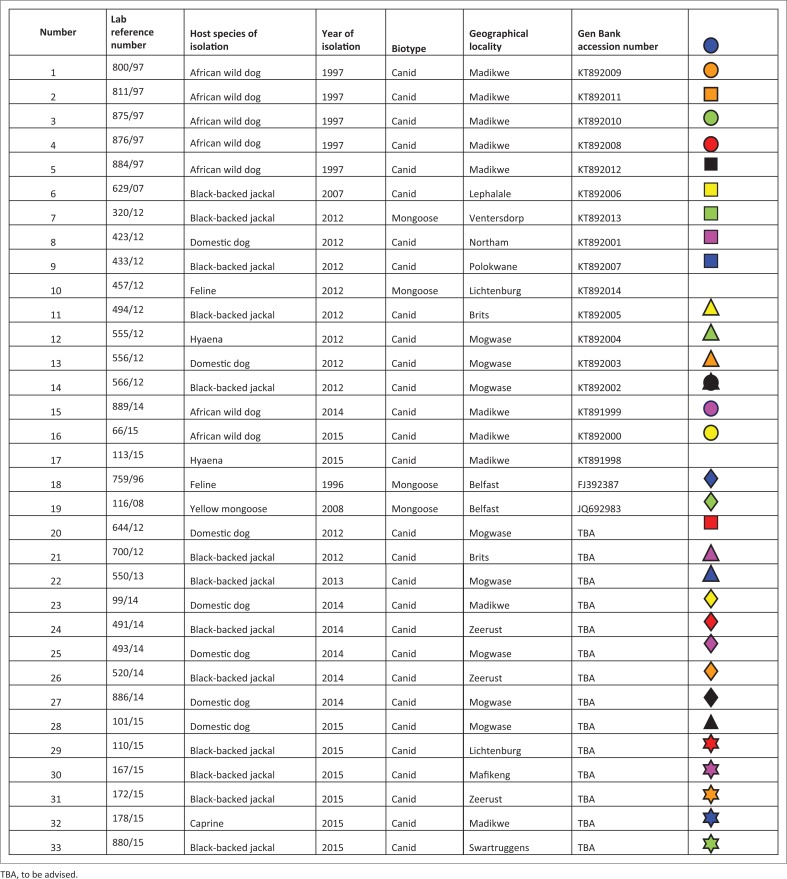
Rabies virus isolates used in the study including wild and domestic animal isolates isolated over a 19-year period.

The lyssaviruses confirmed in the 1997 wild dog rabies outbreak together with the most recent viruses from wild dogs and a hyaena from the Madikwe Game Reserve and adjacent localities were genetically characterised by amplifying and sequencing a partial region of the highly conserved nucleoprotein (N) gene. In brief, Trizol-extracted viral ribonucleic acid (RNA) from original RABV-infected brain tissues (approximately 200 ng of tissue) and freeze-dried infected mouse brain material (for wild dog viruses recovered prior to 2000, see Figure 2) were reverse-transcribed and amplified using the Lys001 (+) (5’-GATTCCTCTCTGCTTCTAG-3’)_1–15_ and 550B (-) (5’-CAAAGGAGAGTTGAGATTGTAGTC-3’)_647–666_ primers (the numbers in subscript indicate annealing positions of the forward and reverse primers in comparison to the Pasteur Virus genome) (Markotter 2007; Sacramento, Bourhy & Tordo 1991). The polymerase chain reaction (PCR) amplicons of approximately 600-bp size were purified using a Qiaquick^®^ PCR purification kit (Qiagen, Germany), sequenced bidirectionally on an ABI3700 (Inqaba Biotech, Pretoria) and consensus nucleotide sequences of both the forward and reverse sequences were generated using algorithms in MEGA 4 (Tamura et al. 2007). A neighbour-joining tree was reconstructed using the nucleotide sequences generated from this study and others from GenBank, which were recovered from domestic and wildlife carnivore species from South Africa (Figure 2). The significance of the branching pattern was statistically evaluated with 1000 bootstrap replicates.

## Results

**FIGURE 3:**
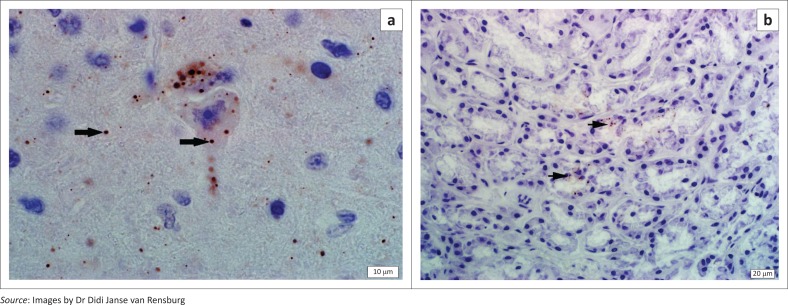
(a) Wild dog hippocampus; rabies antibody immunohistochemical labelling with haematoxylin counterstain (1000× magnification). Viral inclusion bodies, Negri bodies, present within hippocampal neurons and small viral inclusions (rabies dust) present within the neuropil (arrows). (b) Wild dog (case 889/14) mandibular salivary gland, immunohistochemical rabies antibody labelling with haematoxylin counterstain (400× magnification). Numerous small red-brown viral inclusion bodies (arrows) within salivary gland epithelium. This corroborates the known route of transmission via saliva and indicates the advanced stage of infection with rabies virus.

Lyssavirus antigen was confirmed in the brain tissues of the two adult wild dogs and the hyaena using dFAT and for the first wild dog and hyaena using IHC. Typical apple-green fluorescence virus–infected particles were observed in 100% of the field of the composite brain smear and in addition, antigen was observed immunohistochemically in glandular epithelium of the mandibular salivary gland of the first wild dog (889/14) (see Figure 3b). The sections of the brain tissues evaluated showed strong RABV-specific positive immunohistochemical labelling. Numerous, variably sized intracytoplasmic inclusions (Negri bodies and rabies dust) were observed in nerve cell bodies, axons and dendrites in all sections of the brain of both the first wild dog (889/14; Figure 3a) and the spotted hyaena (113/15). The antigenic reactivity patterns of the wild dog and hyaena RABVs were similar and consistent with those observed for other southern African canid RABVs (Ngoepe et al. 2014), commonly maintained in domestic dogs (*Canis familiaris lupus*) and wild carnivore species, such as black-backed jackal (*C. mesomelas*), bat-eared fox (*Otocyon megalotis*) and side-striped jackal (*C. adustus*).

Nucleotide sequencing and phylogenetic analysis of the wild dog RABV partial nucleoprotein gene sequences together with other previously characterised domestic dog and wildlife RABV sequences from South Africa confirmed that canid rabies variant was circulating in these wild dogs (Figure 1). A 100% nucleotide sequence identity of the wild dog RABVs (laboratory reference numbers 889/14 and 66/15) with that recovered from the spotted hyaena (laboratory reference number 113/15) (see Figure 1; alignment matrix not shown) shows the origin as well as a common RABV variant circulating in the wildlife population in this region. The three most recent RABVs from the wild dogs and a hyaena from Madikwe Game Reserve were part of a clade (group 1b) that was closely related to RABVs involved in a rabies outbreak that occurred in the same region but outside Madikwe Game Reserve in the North West province (Figure 3) in 2012 (group 1b) involving hyaena, a black-backed jackal and domestic dogs from Brits and Mogwase. This group (1b) was closely linked to another clade (group 1a) composed of a caprine, jackal and dog viruses.

## Discussion

Rabies trends in the North West province of South Africa over a 10-year period (2005–2015) showed that significant numbers of cases, for example, 44.4% (2007), 58.8% (2012) and 18.3% (2014) of the total annual confirmed animal cases, were positive wildlife cases as recorded at the OIE Rabies Reference Laboratory at the Onderstepoort Veterinary Institute. Whereas the RABVs from wildlife species such as jackals and hyaena in 2007 were of the mongoose rabies biotype, those in 2012 and 2014 were of the canid rabies biotype (Onderstepoort Veterinary Institute records), highlighting the biological variations within the classical RABV variants found in southern Africa and the occurrence of spillover events between wildlife and domestic host species (Nel et al. 2005). A more detailed analysis of the positive rabies cases by species for the period 2012–2015 (Figure 5, as performed by author CS) showed rabies incidence in domestic canines, jackals and bovines of 28.6%, 14.3% and 12.0% of the total positive rabies cases, respectively. A decrease in positive rabies cases in domestic canines and jackal species was observed in 2013. However, in the subsequent year (2014), there was a dramatic increase in positive cases in canines (to 25.0%), bovines (to 46.7%) and jackals (to 8.3%) and similarly in 2015, with increases in canines (to 31.3%) and jackals (to 15.6%), but with an observed decrease in bovine cases (to 28.1%).

**FIGURE 4:**
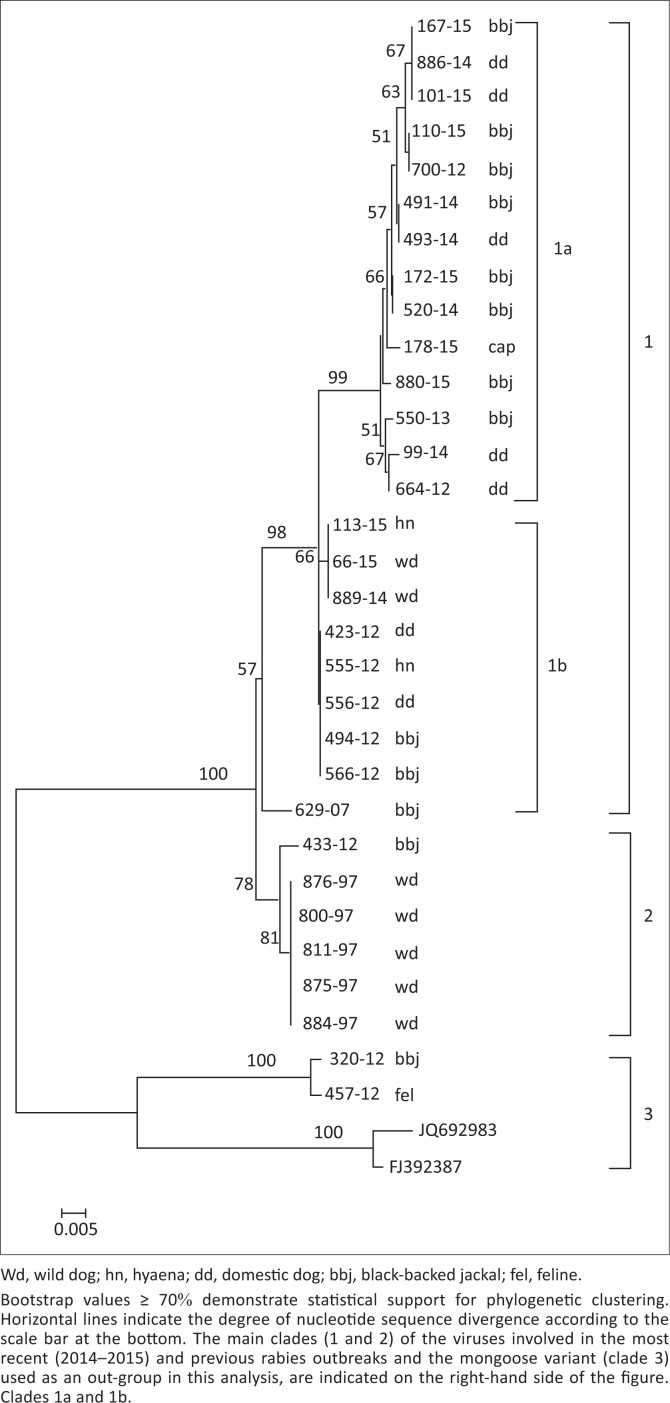
A phylogenetic tree showing the genetic placement and relationships of the recent wild dog rabies virus (clade 1) with rabies virus from other wild carnivores and domestic dogs associated with rabies outbreaks in the North West province.

**FIGURE 5:**
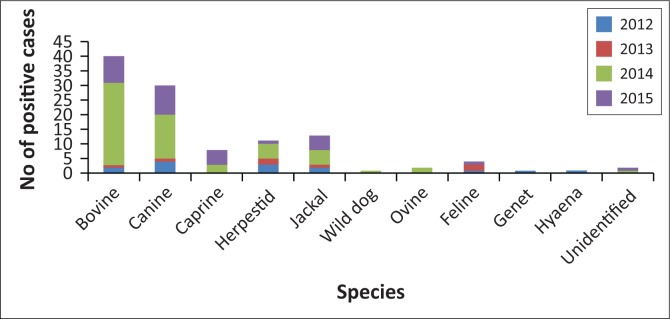
Numbers of positive rabies cases in various species from 2012 to 2015 from the North West province.

Utilising primary diagnostic tests such as the dFAT and IHC, we were able to confirm lyssavirus infection in two wild dogs and a hyaena from Madikwe. The lyssaviruses identified in the current 2014–2015 outbreak inside the park were canid rabies biotype, which delineated into clade 1b of two sub-clusters, 1a and 1b, both consisting of a mixture of domestic (dog) and wildlife (jackal) viruses (Figure 1). This strongly suggested a link between wildlife and domestic dog rabies cycles in this part of South Africa, and such an association is not unique to this region. The clade 1b canid-type rabies isolated was closely related with that diagnosed in dogs, jackals and hyaena outside Madikwe in 2012. Viruses in both clades 1 and 2 could be clearly distinguished from viruses in group 3, representing the mongoose rabies biotype, a variant believed to have been introduced into this region at least 200 years ago (Van Zyl, Markotter & Nel 2010).

In Madikwe Game Reserve in 1997 (Hofmeyr et al. 2000) and again in 2000 (Hofmeyr et al. 2004), it was accepted that a typical jackal virus strain was responsible for the rabies outbreaks. A subsequent report on the molecular epidemiology of rabies in black-backed jackals and dogs in the northern regions of South Africa discovered in the western Limpopo province a jackal-specific rabies cluster which persisted for 5 years, suggesting that the jackal population maintained the rabies cycle in this part of the country (Zulu et al. 2009). The RABV variant responsible for the 1997 outbreak in wild dogs appears to have disappeared and all subsequent wild dog introductions into Madikwe Game Reserve were vaccinated against rabies. A policy was adopted to vaccinate wild dogs whenever an opportunity arose, such as when relocating or darting them (Hofmeyr et al. 2004); however, this was not implemented after 2003. The second outbreak of rabies in the wild dog in Madikwe Game Reserve in 2000 demonstrated the efficacy of vaccination given that none of the five previously vaccinated wild dogs succumbed to RABV infection. It was considered desirable to vaccinate each dog at least twice and this outbreak showed the protective efficacy against natural challenge of multiple vaccination in wild dogs. The RABV neutralising titres obtained after vaccinating wild dogs with an inactivated rabies vaccine (Rabisin, Merial) were therefore significant.

According to Haydon et al. (2002), the target populations of concern in the current outbreak would have been wild dogs and the hyaena, although non-target susceptible species were also considered as potential reservoirs. The question then arose as to the likely sources or reservoirs of infection in the 2014–2015 Madikwe outbreak. The diagnostic and molecular data obtained were insufficient to unequivocally implicate any species, but domestic dogs and cats, jackal, wild dog, hyaena or even bat-eared foxes from inside the park or the same species except wild dogs from outside the park, could potentially have been sources.

In South Africa, ‘predator proof’ fencing around small reserves has been very effective at keeping wild dogs confined inside the reserves, provided the fence is properly maintained, but such fencing has also been reported not to be 100% effective (Davies-Mostert, Mills & Macdonald 2009). The fence surrounding the Madikwe Game Reserve is meant to be continuously electrified, although interruptions do occasionally occur. The fence has an apron packed with rocks to prevent animals excavating below and is composed of large-size mesh (Bonnox [Pty] Ltd, Fencing Manufacturers, Centurion, Pretoria, South Africa), through which only small mammals might pass. Aardvark (*Orycteropus afer*) occasionally dig holes under the fence at Madikwe, although these holes are usually closed by park staff within 24 h of initiation.

Only 2 out of 39 radio-collared jackals managed to pass through the fence to the outside of Madikwe, using holes made by aardvarks (*O. afer*), during six years of research (project field data of RH-W). One of these radio-collared individuals attempted unsuccessfully to regain access into Madikwe despite prolonged movements along the boundary fence. There is therefore a low probability that jackals and/or domestic dogs gain access into Madikwe through the perimeter fence.

In the past 15 years, there has been no record or memory of free-roaming foreign domestic dog sightings within the park. Occasionally, however, domestic cats have been seen and were immediately euthanised by park officials, but not submitted for rabies testing. Cats appear unlikely as a source of RABV infection but cannot be ruled out. Between 2010 and 2016, six cats from the North West province but outside of Madikwe Game Reserve were found to be lyssavirus positive by the OIE Rabies Reference Laboratory, Onderstepoort Veterinary Institute, but only one of these was typed further and found to be mongoose biotype, as were the two feline cases in Figure 2, one of which was from North West province (Lichtenburg, also outside of Madikwe Game Reserve).

Pet dogs belonging to park personnel in Madikwe are supposed to remain enclosed within fencing at staff housing sections; however, occasionally these dogs do escape temporarily into the park. However, all these dogs were vaccinated annually by a veterinarian, including for rabies, with certificates kept up to date. The outlying domestic dog and cat population, for example, those in Molatedi village which lies close to Madikwe, and a subsistence farmer known to have dogs living adjacent to the southern boundary fence should have had their dogs vaccinated as part of a campaign driven by the state; however, on enquiry, vaccination records were not available.

Based on the preceding information, transmission of RABV from jackals to other wildlife carnivores within Madikwe is more likely than from rabid animals occurring outside of Madikwe. Although territorial jackals are generally more spatially confined than dispersing jackals, they have been regularly recorded in Madikwe to travel straight line distances in excess of 8 km from core areas to seek food and water resources. In contrast, dispersal and floater jackals have been recorded to wander over the whole of Madikwe in their quest to seek those areas of least competition from dominant jackals for space and food resources and to facilitate breeding opportunities (field records of RH-W). In this respect, where wild carnivores like lions, hyaenas or wild dogs have made substantial-sized kills, jackals are the most frequently observed mammalian scavengers (field data of RH-W, Madikwe). In Etosha National Park, jackals are the most frequently observed scavengers at anthrax-confirmed carcasses (Bellan et al. 2012). Jackals seek out and are attracted to lion kills, which represent a significant food source for this species in Madikwe (field data of RH-W). Aggressive contacts between jackals that tend to congregate under these circumstances would favour rabies transmission. Although vicious contact confrontations are generally restricted to territorial individuals, conflicts with subordinate dispersing jackals that have been recorded to travel in excess of 40 km during one night in Madikwe may encourage the widespread distribution of rabies within the game reserve (RH-W). RABV transmission is likely in such scenarios with spread possible to several bite victims within a short space of time; these animals then disperse over large ranges and so epidemics may spread and be maintained (Bellan et al. 2012).

The large wild dog pack inhabited mainly the eastern region of the Madikwe Game Reserve and always hunted along the eastern boundary fence (unpublished field data of RH-W). A small number of bat-eared foxes were also recorded in the north-eastern region of the park. Aggressive interactions between wild dogs inside the park and canid species outside the park may occur through the fence but had not been recorded up to 2016.

Wild dogs usually engorge themselves on fresh carcass kills, mostly kudu in Madikwe; they then lie bloated a little distance from the carcass remains. Although jackals are generally wary of wild dogs with wild dogs often chasing jackals from kills (field observations of RH-W), the disorientation and aggressive behaviour of jackals in the terminal phases of rabies may encourage close physical contact interactions between such rabid jackals and wild dogs.

A factor of likely significance in the period prior to the 2014 wild dog rabies outbreak in Madikwe was the effect on the wild dog behaviour of the chemical contraception applied by the park in 2014 as a result of their rapidly expanding population. Wild dogs numbered 34–36 in February 2014 and 26 in September 2014, at which time contraception was introduced. Their numbers thereafter dropped to 13 when counted on 16 February 2015 because of the subsequent rabies outbreak (field data of RH-W). The reproductive intervention had resulted in fragmentation of the northern and southern packs and creation of smaller groups, which appeared to increase antagonistic encounters between these splinter groups. These aggressive contact encounters would likely have exacerbated intra-specific, wild dog rabies transmission. Mills (1990, 1993) outlined how the highly social intra-pack behaviour of wild dogs using mutual mouth licking as well as aggressive biting contact would allow rapid rabies transmission in this species.

Between 2008 and 2010, the Madikwe jackal population was drastically reduced as a consequence of the high lion numbers and densities. The latter resulted in significant opportunistic lion predations on jackals and also an inability of jackals to access lion kills, which in turn were devoured within a short space of time. Lion numbers were reduced steadily between 2010 and 2015 as a result of management initiatives. Jackal numbers are positively favoured when lion numbers are low and when the percentage of lions under 2 years in the population is small, as young lions have been regularly recorded to kill jackals (field observation data of RH-W, Madikwe).

On 15 of January 2015, a video was recorded of a wild dog male eating a dead wild dog (data of RH-W), highly likely at that time to have died of rabies. The last wild dog of the larger pack, which was the second positively diagnosed wild dog case, was shot and submitted to Onderstepoort on 26 January 2015.

The devastating effect of rabies on the wild dog population in Madikwe during the latter half of 2014 was not paralleled in the jackal population (data of RH-W), although data suggest that jackal breeding pairs declined by 15.7% between 2013 and 2014 (pre-rabies outbreak) and by a further 6.2% between 2014 and 2015. In this respect, severe mange, small jackal litter sizes, poor jackal pup survivals and a continued lack of good jackal representations at large carcasses and lion kills between 2013 and 2015 implicate the progressively worsening drought conditions and associated food deprivations as pivotal factors in the poor performance of jackals during this period. Although it was not possible in 2014 to confirm whether all adult jackal pairs associated with pup mortalities were alive after the loss of some or all their pups, some jackal pairs were recorded in their territorial areas after these after the death of these pups. These data challenge rabies as an implicating factor in jackal population mortalities during the latter half of 2014, when rabies probably peaked in the Madikwe wild dog population. This hypothesis is further supported by the observed increase in the numbers of jackals reported at large carcasses during 2016, which suggests that jackal pup survival rates from 2015 jackal litters had improved. The increased availability of food from the extraordinarily large amount of ungulate drought-related mortalities that were present in the field during the latter half of 2015 would have favoured jackal pup survival. The quandary with respect to the origins of the rabies outbreak and as to whether there is a possibility that rabies is present and maintained in certain wild species populations as reservoir hosts within Madikwe needs further investigation (field data and interpretation of RH-W).

Spread of rabies amongst members of a spotted hyaena clan as well as inter-clan spread depends on the number of clans, clan size, their density in an area and on the ranks of the affected animals, which would affect behaviour and potential contacts with other hyaenas. In general, widely distributed clans or clans with few members would show slow intra-clan and inter-clan spread. Spotted hyaenas rarely meet with all clan members and individuals tend to be solitary or in small groups. They are more territorial than wild dogs: they scent-mark and defend their territories from other clans (Mills 1993). An unusual rabies outbreak was recorded in southern Kalahari in a small spotted hyaena clan in 1990 where all four clan members died (Mills 1990, 1993), whereas in another southern Kalahari outbreak affecting several clans, none of the packs were decimated. Jackals often interact with hyaena around carcasses and bite contacts may occur between the two species (Mills 1993). Reliable park data on accurate hyaena numbers during the years prior to and during the 2014–2015 Madikwe rabies outbreak were, unfortunately, not available.

In February 2015, the remaining five adult wild dogs and eight pups in Madikwe were vaccinated against rabies but six pups still died, although none were tested for rabies. Wild dog pups in Kruger National Park have been recorded to have a high mortality rate, many because of unknown cause but several by lion predation (Mills 1993). The 2016 Madikwe population figures showed that both wild dog and jackal populations had recovered (data of RH-W).

As human populations increase around reserve borders, the risk to wild dogs which venture outside or escape from parks is also likely to increase. Humans in game parks are at considerable risk of contact with rabid carnivores because several, as in this situation, are shot within tourist lodges or camps or in close proximity to them (Bellan et al. 2012). From 1975 to 2010, laboratory-confirmed rabies was found in 67 jackals and 30 other animals all destroyed in the Okaukuejo tourist camp in Etosha National Park, Namibia (Bellan et al. 2012). Jackals and other species, when healthy and non-rabid, also often eat human food waste near tourist camps and so frequent these areas; in this situation, they may also be shot by park officials.

Contact between some wild carnivores such as jackals and domestic dogs is a common phenomenon in southern Africa and provides an ideal opportunity for pathogen transmission across species boundaries (Bingham 2005; Bingham et al. 1999a; Sabeta, Bingham & Bel 2003), given the opportunistic nature of the canid rabies variant as evidenced in Figure 2. In Zimbabwe, outbreaks of rabies in wild canids such as jackals followed, rather than preceded, outbreaks in domestic dog populations, highlighting that dogs are potential sources of the disease in wildlife (Bingham et al. 1999b). Dogs are known as reservoirs of rabies because they can independently maintain infection within a population (Lembo et al. 2008). However, Bingham et al. (1999a, 1999b) suggested that although jackal infection is initiated by domestic dogs, the infection may then be sustained in jackals over several years. It is likely that rabies in the current study area of Madikwe had a complex multi-host reservoir or transmission system which may have involved domestic dogs (or cats) but more likely involved wild carnivores, especially jackal and possibly bat-eared foxes, although no concrete evidence was found for any specific reservoir. Spillover of rabies from domestic dog populations may initiate short-lived chains of transmission in other carnivores (Lembo et al. 2008). During the current Madikwe outbreak, rabies was confirm in one spotted hyaena and and 24 wild dogs likely to have succumbed to rabies; however, specimens were not submitted for laboratory confirmation from all cases because of carcasses not being found or being too decomposed. A jackal with suspected rabies signs was shot in October 2015 in Madikwe but unfortunately buried; this was several months after the wild dog outbreak.

Molecular epidemiology, the scientific investigation that focuses on the contribution of potential genetic and environmental risk factors, has now become useful in tracing routes and origins of infection, as well as in identifying the emergence of new variants, as exemplified by lyssavirus evolution; a phenomenon illustrated in the current study. A database of nucleotide sequences for the N gene for South African domestic and wildlife carnivore species will be useful in future in order to assess origins and mapping of movement of rabies outbreaks. Similarly, nucleotide sequences of the rabies viral N and glycoprotein (G) genes from virus isolates of four wild dogs (including an individual from Tanzania) indicated that a viral variant common amongst domestic dogs was responsible for RABV infection in wildlife carnivores in Kenya and Tanzania (Kat et al. 1995), underlining the important role that domestic carnivores play in spillover of disease to wildlife carnivore species. In East Africa, similar to and corroborating the results obtained in this study, RABVs recovered from wild dogs in the Serengeti ecosystem, including the Kenyan Masai Mara and Tanzania, were serologically and genetically indistinguishable from the typical domestic dog strain in dogs from their neighbouring communal areas, indicating dogs as the potential reservoir of RABV infection (Kat et al. 1996).

Molecular techniques have been shown to be useful in confirming RABV infection even in decomposed and exhumed carcasses, as recently demonstrated by two independent groups (Markotter et al. 2015; McElhinney et al. 2014). In the former study, severely decomposed brain material was tested for the presence of RABV genomic material using a quantitative real-time reverse transcription–PCR (q-real-time RT-PCR) assay when conventional molecular methods were unsuccessful. In the latter study, carcasses received for rabies diagnosis were occasionally decomposed because of delays in them being found, in submitting or storing them and in such cases a rabies-specific hemi-nested RT-PCR could detect viral RNA on day 70 of the storage of the carcass, suggesting the persistence of infectious RABV in carcasses. The detection of RABV RNA from a carcass decomposing at 35 °C for 48 days supports the use of molecular assays to accompany OIE-prescribed rabies diagnostic tests particularly when decomposed samples are submitted (McElhinney et al. 2014). Therefore, it is important that field personnel be aware of the applicability of the dFAT and molecular-based tests such as PCR in diagnosing lyssavirus infection in decomposed specimens. It should also be remembered that carcasses which have been buried and later exhumed for specimen taking as for rabies may be surprisingly reasonably preserved as a result of the low and constant ambient underground temperature.

The value of IHC for rabies diagnosis in formalin-fixed samples was clearly demonstrated in the current study. If fresh tissues cannot be submitted for whatever reason, as commonly happens in remote areas of South Africa and other parts of the continent, or where other preservatives such as glycerol saline may be in short supply, IHC can be considered as an equivalent alternative method for rabies diagnosis in animal tissues where diagnostic facilities offer such tests. Furthermore, with IHC, the virus inhabiting cells and any associated pathological changes in the brain are clearly observable, in comparison with other diagnostic methods such as the dFAT or molecular techniques. Formalin fixation of the brain preserves tissue architecture and allows histological evaluation to formulate a differential diagnosis (Stein et al. 2010). With IHC, antigen was observed in the salivary gland of the one wild dog, thereby illustrating the advanced clinical stage of infection in this animal. Thus, IHC provides a diagnostic alternative to dFAT and can be used with confidence – specificity and sensitivity of rabies IHC is currently being formally investigated (to be published, author DJvR); however, our experience shows it to be as sensitive as the dFAT. Immunohistochemistry is of use particularly in tropical and remote areas where cold storage and rapid transport of fresh tissues may be impossible, and where exposure to fresh sample tissue may place human or animal contacts under zoonotic risk.

Personnel and laboratory employees should treat all fresh samples from suspect rabies cases as potentially rabies-infected and when appropriate throughout the dFAT procedure, as RABV is not inactivated with acetone fixation (Jarvis, Franke & Davis 2016). Exposure of fresh and infective tissue to 2% buffered formaldehyde for 60 min inactivated vaccinia virus, human adenovirus and murine norovirus into non-infectious but structurally intact forms allowing for morphological diagnosis by electron microscopy (Möller et al. 2015), with effectivity increasing at higher temperatures of 25 °C – 37 °C. Another study reported that 3% formalin (1% formaldehyde) was ineffective in inactivation of 99% of 10 000 mouse intracerebral lethal dose, 50% (LD_50_) of RABV within 1.0–2.5 min (Kaplan, Wiktor & Koprowski 1966). It is generally accepted that 10% neutral buffered formalin preserves tissues and inactivates most pathogens including viruses but excluding prions, at room temperature and when left for at least 24 h – 48 h; however, specific reference to this was not found. The optimal ratio of the tissue:formalin is 1:10, and bulky firm tissues should be incised at regular 1-cm or narrower intervals to allow penetration of the formalin.

## Conclusion

In conclusion, in order for effective control of rabies outbreaks in domestic mammals, non-endangered and endangered wildlife and humans, the epidemiology of RABV infection in these species and in varying circumstances requires ongoing intensive study. This implies that study of predator interactions, movements, behaviour, food sources, competition and the dynamics which alter with human interventions and natural environmental changes within the confines of game parks should be ongoing. Close monitoring of species numbers and submission of material for diagnosis when carcasses are found or when animals are euthanised as a result of aberrant behaviour are essential. Because of its multi-host nature, RABV is an important threat to ecologically threatened animal populations such as the wild dog. RABV is a pathogen that can be effectively controlled or eliminated at the primary host animal source by vaccination. Parenteral vaccination is key to the potential elimination of some viral diseases from domestic dogs and cats and further mitigates transmission of rabies and other pathogens to both wildlife and humans. The diligent ongoing implementation of suitable, effective vaccination of wild carnivores against RABV for continued prevention of the disease and the preservation of these species, although controversial, is recommended.
